# Age- and sex-dependent role of osteocytic pannexin1 on bone and muscle mass and strength

**DOI:** 10.1038/s41598-019-50444-1

**Published:** 2019-09-25

**Authors:** Alexandra Aguilar-Perez, Rafael Pacheco-Costa, Emily G. Atkinson, Padmini Deosthale, Hannah M. Davis, Alyson L. Essex, Julian E. Dilley, Leland Gomez, Joseph E. Rupert, Teresa A. Zimmers, Roger J. Thompson, Matthew R. Allen, Lilian I. Plotkin

**Affiliations:** 1 Department of Anatomy, Cell Biology & Physiology, Indiana University School of Medicine, Indianapolis, IN 46202 USA; 2 Roudebush Veterans Administration Medical Center, Indianapolis, IN 46202 USA; 3 Indiana Center for Musculoskeletal Health, Indianapolis, IN 46202 USA; 4 Department of Surgery, Indiana University School of Medicine, Indianapolis, IN 46202 USA; 5 Indiana University Simon Cancer Center, Indianapolis, IN 46202 USA; 6 Hotchkiss Brain Institute, Department of Cell Biology and Anatomy, University of Calgary, Calgary, Alberta Canada

**Keywords:** Cell death, Mechanisms of disease

## Abstract

Pannexins (Panxs), glycoproteins that oligomerize to form hemichannels on the cell membrane, are topologically similar to connexins, but do not form cell-to-cell gap junction channels. There are 3 members of the family, 1–3, with Panx1 being the most abundant. All Panxs are expressed in bone, but their role in bone cell biology is not completely understood. We now report that osteocytic Panx1 deletion (Panx1^Δot^) alters bone mass and strength in female mice. Bone mineral density after reaching skeletal maturity is higher in female Panx1^Δot^ mice than in control Panx1^fl/fl^ mice. Further, osteocytic Panx1 deletion partially prevented aging effects on cortical bone structure and mechanical properties. Young 4-month-old female Panx1^Δot^ mice exhibited increased lean body mass, even though pannexin levels in skeletal muscle were not affected; whereas no difference in lean body mass was detected in male mice. Furthermore, female Panx1-deficient mice exhibited increased muscle mass without changes in strength, whereas Panx1^Δot^ males showed unchanged muscle mass and decreased *in vivo* maximum plantarflexion torque, indicating reduced muscle strength. Our results suggest that osteocytic Panx1 deletion increases bone mass in young and old female mice and muscle mass in young female mice, but has deleterious effects on muscle strength only in males.

## Introduction

Pannexin 1 (Panx1) is the most widespread member of the pannexin family of proteins and its mRNA expression has been detected in all bone cells^1–3^. Similar to connexins, Panx1 molecules associate to form hemichannels in the cell membrane, but unlike connexins, there is no evidence for the presence of pannexin channels linking neighboring cells^4,5^. Global Panx1-deficient mice have been generated and Panx1 deficiency does not alter cancellous bone structure in the distal femur, when compared to wild type mice^6^. However, fatigue loading-induced increased intracortical resorption observed in wild type is absent in Panx1-deficient mice^6^. Whether deletion of Panx1 alters bone mass or mechanical properties during aging has not been reported.

Panx1 channels are permeable to ATP and open in response to various stimuli including mechanical stress, Panx1 ligands, low oxygen concentration, increased K^+^ and intracellular calcium levels, and apoptosis^7^. In particular, activation of caspase-3 in apoptotic cells results in cleavage of the Panx1 auto-inhibitory C-terminus tail, irreversibly opening the channels^8^. Indeed, he increase in selective membrane permeability that occurs in the early stages of apoptosis has been ascribed to Panx1 channel opening. Small molecules are then released through Panx1 channels, including nucleotides. ATP, one such nucleotide, can then act as a chemotactic signal to recruit cells with phagocytic activity^9^.

Osteocytes, considered the main regulatory cells in bone, produce and release molecules that modulate the differentiation and activity of osteoblasts and osteoclasts, thereby controlling bone modeling and remodeling^10^. Further, apoptotic osteocytes are believed to recruit osteoclasts, leading to targeted bone resorption^11^. There is also evidence that osteocyte viability influences bone mechanical properties, with increased osteocyte apoptosis in conditions with increased bone fragility; conversely, reduced osteocyte apoptosis is associated with treatments that increase bone mechanical properties^12^. More recently we have reported that apoptotic osteocytic cells release factors to the culture media (conditioned media) *in vitro* that stimulate osteoclastogenesis; and that prevention of osteocytic cell apoptosis reversed the increase in osteoclast formation induced by the conditioned media from osteocytic cells^13^. Further, there is higher osteoclast inducing activity in the conditioned medium of bones from old mice (21-months) than from young mice (4-months). Treating these bones with an apoptosis inhibitor reduced both apoptosis and osteoclast-inducing activity^14^. Taken together, these pieces of evidence suggest that osteocyte apoptosis leads to the release of osteoclastogenic factors. This led us to propose that, with aging and increased osteocyte apoptosis, Panx1 channels open and release factors that increase osteoclastogenesis and ultimately, bone resorption.

To test the potential role of Panx1 channels in skeletal aging, we generated mice lacking Panx1 in cells expressing the 8 kb fragment of the DMP1 promoter, which we previously showed deletes genes preferentially from osteocytes^15–18^. We found that Panx1 deletion has small but significant effects on bone mass that is more evident as the mice age. Further, absence of Panx1 prevented the increase in apoptosis-associated genes and the decrease in bone strength detected even in the relatively young 13-month-old mice. Unexpectedly, osteocytic Panx1 deletion augmented muscle mass in young female mice, but not in male mice, even though Panx1 was not deleted from skeletal muscle in our model. Our results suggest that osteocytic Panx1 can mediate the effect of osteocytes as regulators of bone and muscle mass in an age- and sex-dependent manner.

## Results

### Prevention of apoptosis and maintenance of bone mass in middle aged mice lacking Pannexin 1 in osteocytes

**Figure 1:**
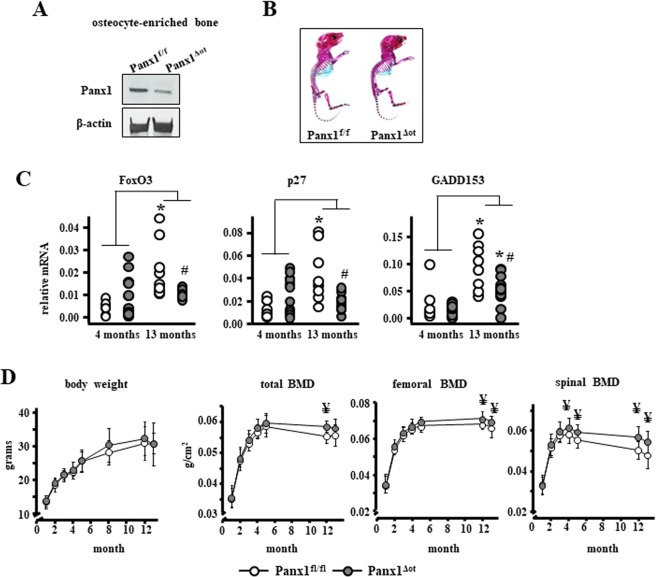
Deletion of Panx1 in osteocytes increases bone mass. (**A**) Panx1 protein levels in calvaria bones from Panx1^fl/fl^ and Panx1^Δot^ mice at 4 months of age were assessed by Western blotting and β-actin was used as a loading control. (**B**) Representative images of whole body histological preparations (n = 3/genotype) stained by Alcian blue/alizarin red in 6-day-old mice for visual inspection of cartilage (blue) and calcified tissue (magenta). (**C**) The levels of the apoptosis-related genes were measured by qPCR bone marrow-flushed long bones obtained from female mice. Bars represent mean ± s.d., ^#^p < 0.05 versus Panx1^fl/fl^ mice at the same age, *p < 0.05 versus 4-month-old mice of the same genotype, and lines indicate age effect, by 2-way ANOVA. (**D**) Body weight and total body, spinal and femoral BMD were assessed monthly in female mice from 1 to 13 months of age by DXA (n = 9–12 per group). ^¥^p < 0.05 versus Panx1^fl/fl^ mice at the same age by t-test.

We examined tissues from mice carrying the DMP1-8kb-Cre transgene and Panx1 floxed alleles (Panx1^ΔOt^ mice) and littermate mice bearing only floxed Panx1 (Panx1^fl/fl^). Panx1 protein levels were decreased by 50% in calvaria bone from female Panx1^ΔOt^ mice, compared to control Panx1^fl/fl^ mice (Fig. 1A). Mice lacking Panx1 in osteocytes did not exhibit apparent anatomical abnormalities and qualitatively showed similar distribution of cartilage and mineralized bone at birth as Panx1^fl/fl^ littermates (Fig. 1B). Aging resulted in increased mRNA expression of apoptosis-associated genes Foxo3, Cdkn1b/p27, and Ddit3/GADD153 in cortical bone preparations containing osteocytes from control Panx1^fl/fl^ mice (Fig. 1C), consistent with the increase in TUNEL positive osteocytes documented in 14-month-old wild type mice^19^. Unexpectedly, deletion of Panx1 from osteocytes prevented the increase in all apoptosis-associated genes tested in female mice. However, since the gene expression analysis was performed using whole tissue, it is not possible to conclusively determine which cell type is undergoing apoptosis.

Longitudinal analysis of body weight showed no differences between genotypes in female mice up to 13 month of age (Fig. 1D). On the other hand, Panx1^ΔOt^ mice exhibited higher total BMD (5.5%) at 12 months of age, compared to age- and sex-matched Panx1^fl/fl^ littermate mice. Femoral BMD was higher (5.6–13.9%) at 12 and 13 months of age in Panx1^ΔOt^ compared to Panx1^fl/fl^ littermates, and spinal BMD (3.2–5.1%) was higher starting at 5 months of age.

**Figure 2:**
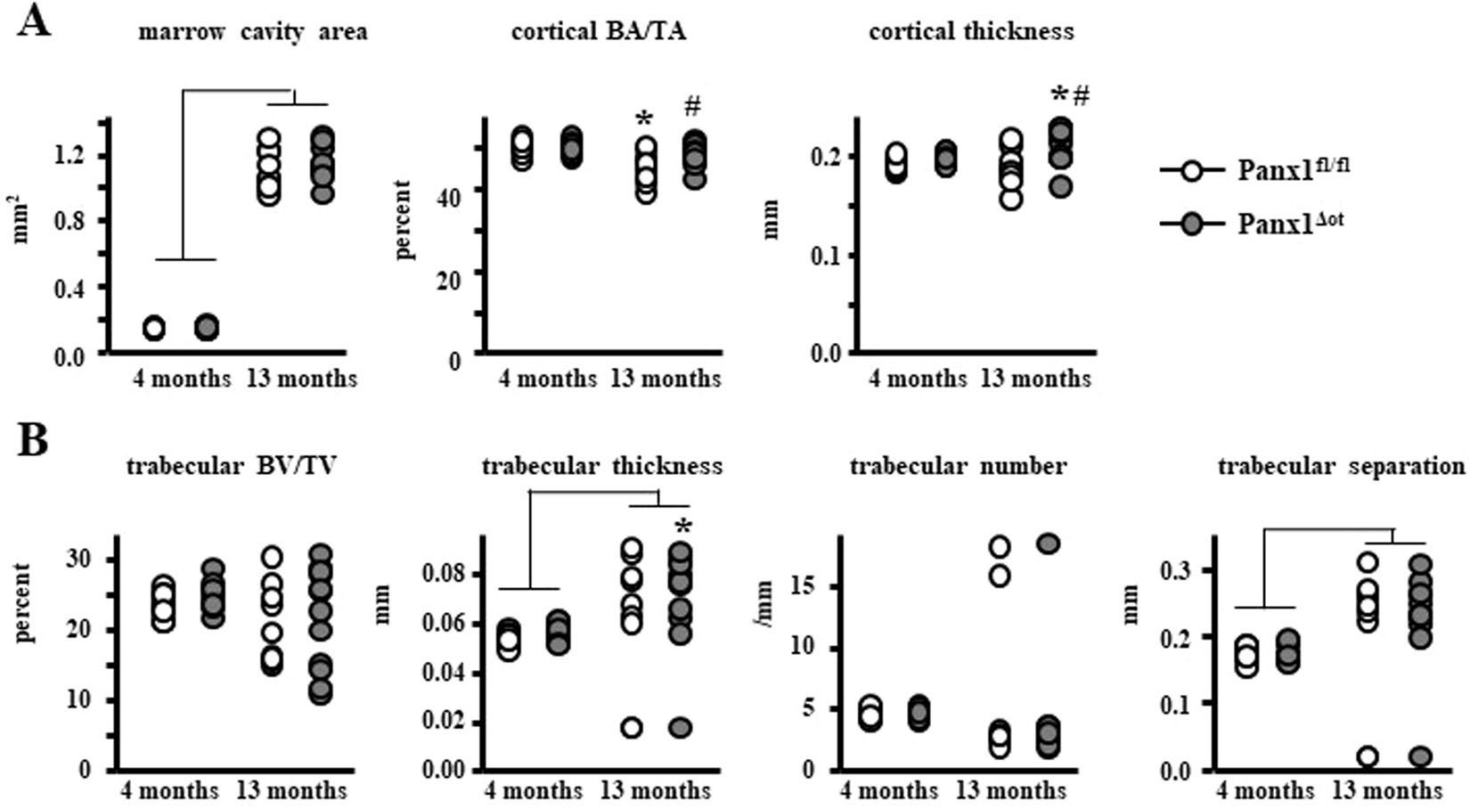
Osteocytic Panx1 removal preserves cortical bone volume and increases cortical thickness in aged female mice. (**A**) Femur cortical bone geometry in the mid-diaphysis was assessed by µCT (n = 9–12 per group). (**B**) Analysis of cancellous bone microarchitecture in L5 vertebra was assessed by µCT (n = 9–12 per group). Bars represent mean ± s.d., ^#^p < 0.05 versus Panx1^fl/fl^ mice at the same age, *p < 0.05 versus 4-month-old mice of the same genotype, and lines indicate age effect, by 2-way ANOVA.

We next examined the structural and cellular effects of Panx1 deletion in 4- and 13-month-old female mice. Four-month-old C57BL/6 mice are considered mature adults (equivalent to a 20/30-year-old human), whereas 13-month-old mice are considered middle-aged (equivalent to 38/47-year-old human)^20^. We selected these two ages because there was a significant difference in BMD between those 2 ages in control mice, and a significantly higher BMD in the Panx1^ΔOt^ mice compared to Panx1^fl/fl^ mice at 13 months. μCT analysis of the femoral mid-diaphysis revealed that aging resulted in a larger marrow cavity area, independent of Panx1 expression, lower cortical bone area/tissue area only in control Panx1^fl/fl^ mice, and higher cortical thickness only in Panx1^ΔOt^ mice (Fig. 2A). Panx1 deletion prevented the decrease in cortical bone area/tissue area and increased cortical thickness in the femoral mid-diaphysis in the aged animals. In the lumbar vertebrae, the overall aging effect was only manifested by an increase in trabecular separation, independent of Panx1 (Fig. 2B). Further, aged Panx1^ΔOt^ mice exhibit increased trabecular thickness compared to young Panx1^ΔOt^.

### Improved mechanical properties in the absence of Panx1 in osteocytes

**Figure 3:**
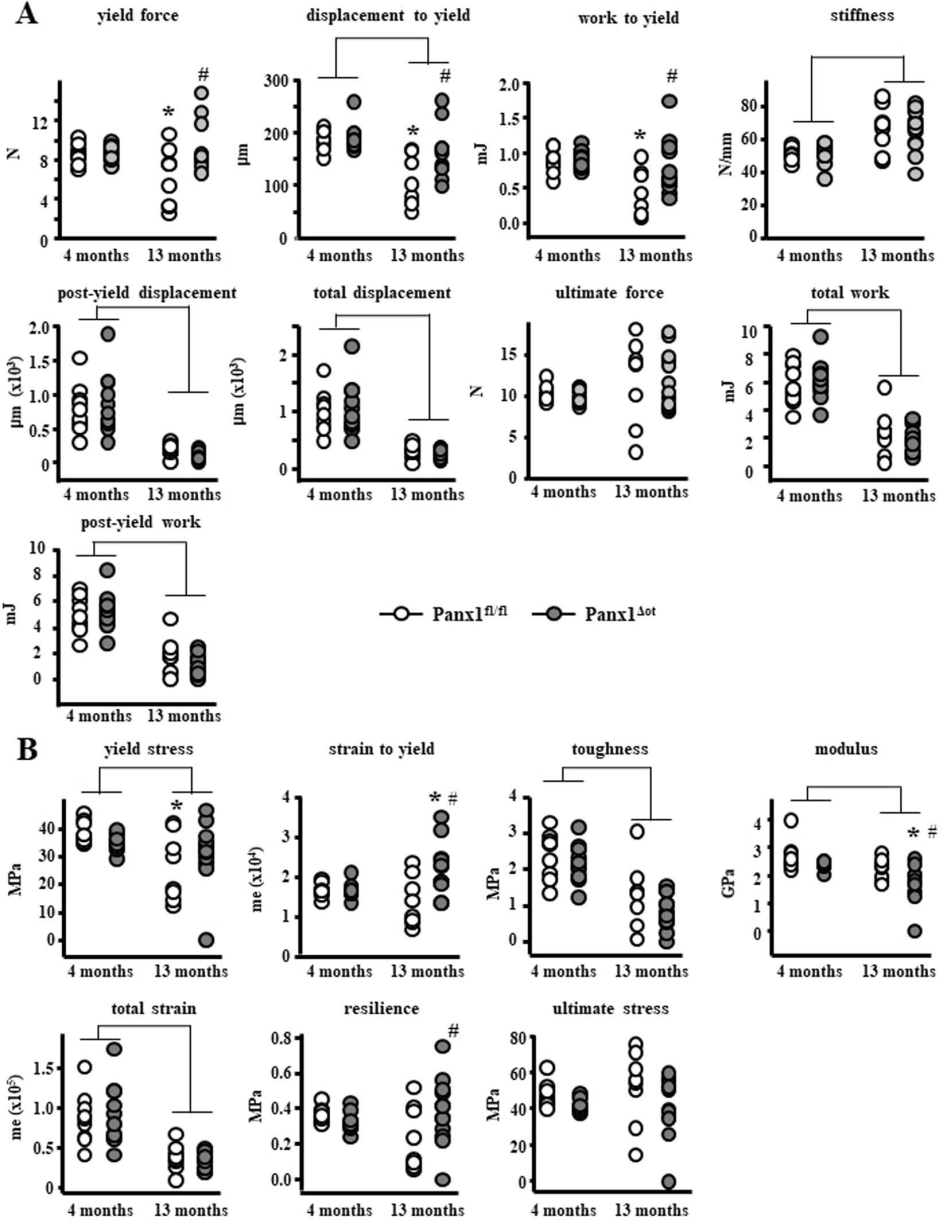
Aged Panx1^Δot^ female mice have increased mechanical properties in cortical bone. Femur cortical bone structural (**A**) and material (**B**) mechanical properties were measured by 3-point bending at 4 and 13 months of age female mice. (n = 9–12 per group). Bars represent mean ± s.d., ^#^p < 0.05 versus Panx1^fl/fl^ mice at the same age, *p < 0.05 versus 4-month-old mice of the same genotype, and lines indicate age effect, by 2-way ANOVA.

In control Panx1^fl/fl^ female mice, mechanical testing of the femoral mid-diaphysis revealed a significant overall decrease in the structural mechanical displacement and work parameters as well as increased stiffness in 13-month-old compared to 4-month-old female mice (Fig. 3A). In addition, we found an age-related effect on the material-level mechanical properties yield stress, toughness, modulus, and total strain, which were all significantly reduced (Fig. 3B). This evidence indicates that even though the 13-month-old are not considered “old”, there is a substantial deterioration of the cortical bone mechanical properties at both the structural and material level. Deletion of Panx1 in Panx1^ΔOt^ mice preserved some of those properties, yield force, work to yield, and yield stress were only decreased in aged Panx1^fl/fl^ mice, compared to 4-month-old mice of the same genotype. Further, deletion of Panx1 resulted in increased yield force, displacement to yield, work to yield, strain to yield and resilience in the Panx1^ΔOt^ compared to Panx1^fl/fl^ mice at 13 months of age. On the other hand, modulus was only decreased in 13-month-old Panx1-deficient mice compared to mice of the same genotype at 4 months of age, and to Panx1^fl/fl^ mice at 13 months of age. This evidence suggests that the presence of Panx1 in osteocytes in aged mice is deleterious for bone biomechanical properties and that by deleting the gene; some mechanical properties are improved to levels that, in some cases, are similar to those of younger animals.

### Osteocyte Pannexin depletion does not alter bone formation or osteoclast number/surface in young or aged mice

**Figure 4:**
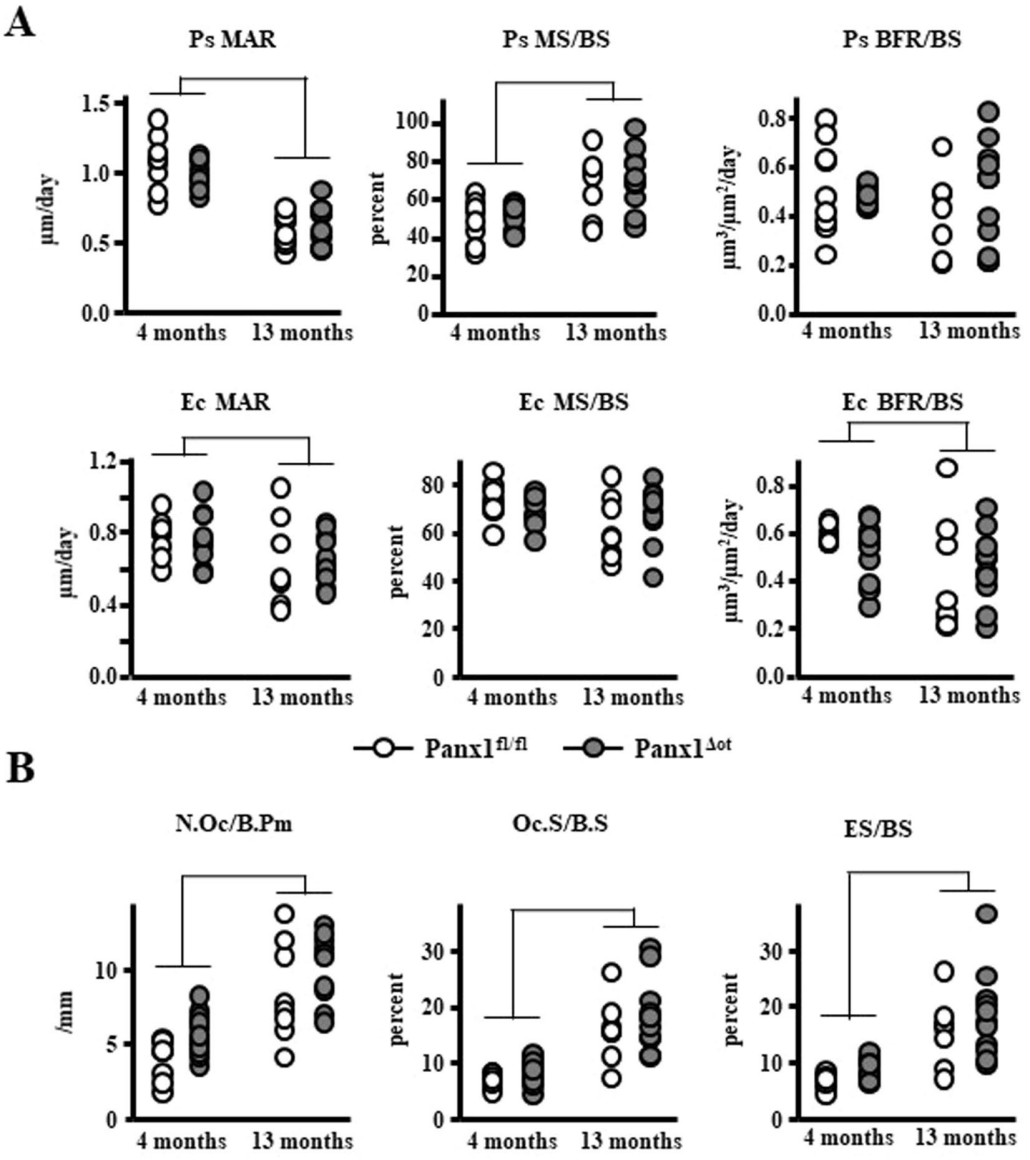
Panx1 deletion does not alter bone formation or osteoclast number/surface in cortical bone from female mice. (**A**) Periosteal (Ps) and endocortical (Ec) MS/BS, MAR, and BFR/BS were measured in unstained femoral cross-sections (n = 9–12 per group). (**B**) N.Oc/BS, Oc.S/BS, ES/BS were scored on the endocortical surface of the femoral mid-diaphysis after staining for TRAPase/Toluidine blue in Panx1^fl/fl^ and Panx1^Δot^ mice at 4 and 13 months of age (n = 9–12 per group). Bars represent mean ± s.d., Lines indicate age effect, by 2-way ANOVA.

Consistent with our previous studies^19^, 13-month-old mice exhibited a decrease in mineral apposition rate and an increase in mineralizing surface on the periosteal surface of the femoral mid-diaphysis, the net effect being no difference in bone formation rate (Fig. 4A). Mineral apposition rate was also decreased on the endocortical surface of the femur, whereas mineralizing surface was not affected, resulting in a decrease in bone formation rate in aged mice. In addition, osteoclast number and surface, as well as eroded surface were all increased on the endocortical surface of aged mice, independently of whether they express Panx1 or not (Fig. 4B). Two-way-ANOVA analysis of the parameters revealed no effect of genotype and no age-genotype interactions (Table 1). Our data suggests that overall; deletion of Panx1 from osteocytes did not alter histomorphometric parameters in the cortical bone of the femoral mid-diaphysis.

**Table 1:** p values for age, genotype, and their interaction by 2-way ANOVA.

Figure	endpoint	p value		
		age	genotype	age x genotype
1C	FoxO3 mRNA	**0.002**	0.443	**0.004**
	p27	**0.017**	0.166	**<0.001**
	GADD153	**<0.001**	**0.007**	0.079
2A	marrow cavity area	**<0.001**	0.417	0.506
	BA/TA	<0.001	0.060	0.067
	cortical thickness	0.309	**0.006**	0.055
2B	BV/TV	0.074	0.436	1.891
	Tb.Th	**0.030**	0.281	0.548
	Tb.N	0.747	0.505	0.479
	Tb.Sp	**0.044**	0.590	0.576
3A	yield force	0.145	**0.020**	**0.029**
	displacement to yield	<**0**.**001**	**0.015**	**0.039**
	work to yield	**0.002**	**0.030**	0.052
	stiffness	**<0.001**	0.862	0.895
	post-yield displacement	**<0.001**	0.796	0.521
	total displacement	**<0.001**	0.954	0.718
	ultimate force	0.072	0.933	0.987
	total work	**<0.001**	0.841	0.396
	post-yield work	**<0.001**	0.468	0.170
3B	yield stress	**0.005**	0.800	0.100
	strain to yield	0.419	0.010	0.009
	toughness	**<0.001**	0.124	0.682
	modulus	**<0.001**	0.006	0.289
	total strain	**<0.001**	0.908	0.837
	resilience	0.441	0.210	**0.037**
	ultimate stress	0.508	0.080	0.576
4A	Ps MAR	**<0.001**	0.607	0.163
	Ps MS/BS	**<0.001**	0.563	0.819
	Ps BFR/BS	0.352	0.740	0.342
	Ec MAR	**0.017**	0.929	0.757
	Ec MS/BS	0.085	0.676	0.112
	Ec BFR/BS	**0.025**	0.751	0.257
4B	Oc.N/B.Pm	**<0.001**	**0.023**	0.993
	Oc.S/BS	**<0.001**	0.127	0.654
	ES/BS	**<0.001**	0.119	0.826
5A	MAR	**<0.001**	**0.003**	0.110
	MS/BS	0.958	0.638	0.141
	BFR/BS	**<0.001**	**0.012**	0.205
5B	Oc.N/B.Pm	0.814	0.108	**0.017**
	Oc.S/BS	**<0.001**	0.159	0.764
	ES/BS	**<0.001**	0.246	0.439
5C	N.Ob/BPm	**<0.001**	0.363	0.808
	Ob.S/BS	**<0.001**	0.345	0.742
5D	P1NP	**<0.001**	0.182	0.239
	ALP	**0.001**	0.433	0.806
6A	CD11b	0.666	**0.021**	0.207
6B	Rankl	**0.002**	**0.027**	**0.002**
	OPG	**<0.001**	0.214	0.059
	Rankl/OPG	**0.001**	0.786	0.787
6C	TRAP	0.060	0.110	**0.007**
	DCSTAMP	**<0.001**	0.014	0.004
	NFATC1	**<0.001**	0.052	**0.032**

**Figure 5:**
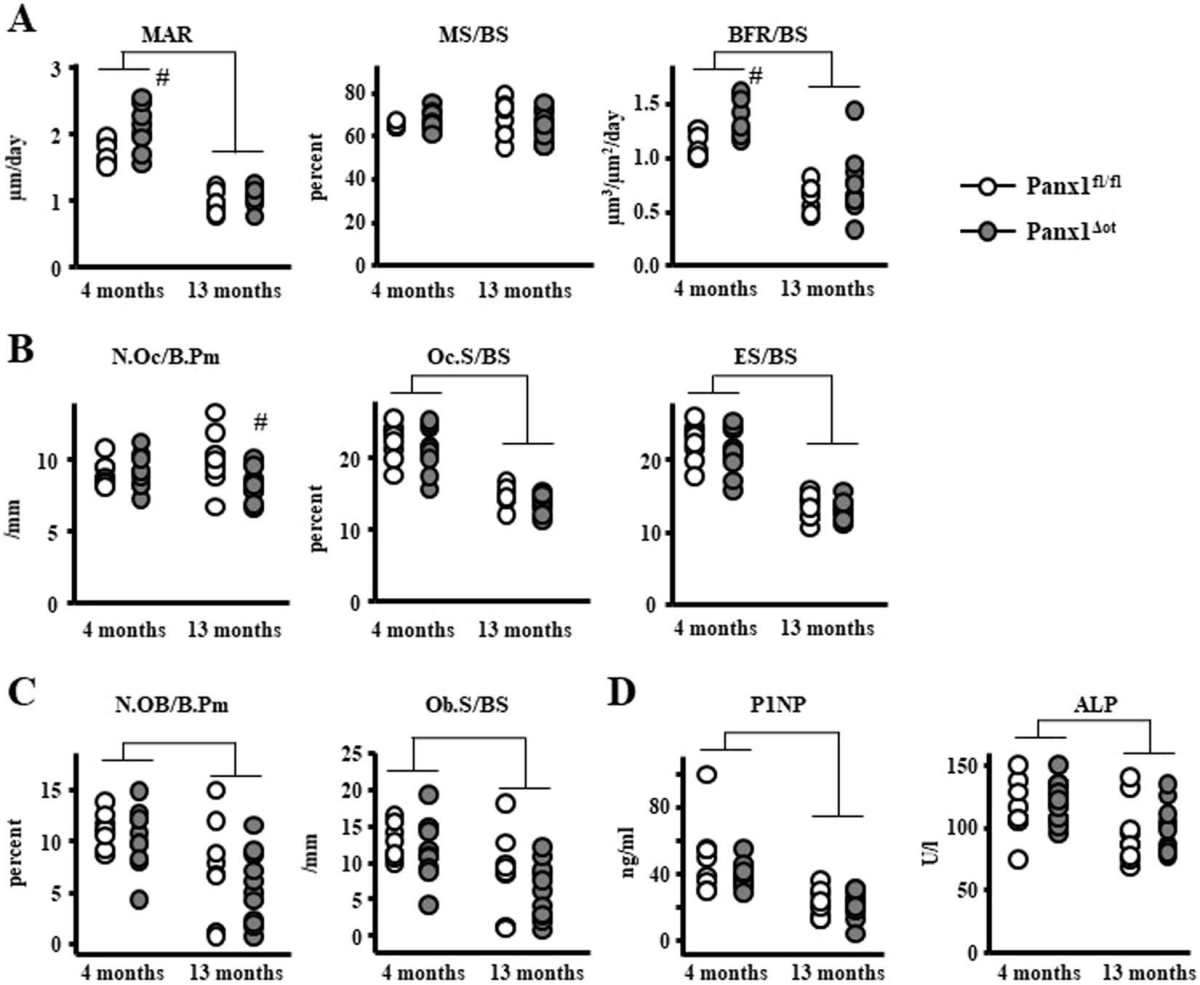
Panx1^Δot^ female mice exhibited minimal changes in vertebral dynamic and static histomorphometric parameters. (**A**) MS/BS, MAR, and BFR/BS were measure in unstained sections of lumbar vertebra (n = 9–12 per group). (**B**) N.Oc/BS, Oc.S/BS, ES/BS were scored in vertebral cancellous bone after staining for TRAPase/Toluidine blue in Panx1^fl/fl^ and Panx1^Δot^ mice at 4 and 13 months of age (n = 9–12 per group). (**C**) N.Ob/BS and Ob.S/BS were scored in lumbar vertebra stained with von Kossa/McNeal (n = 9–12 per group). (**D**) Circulating markers of bone formation (P1NP, 9–12 per group and ALP, n = 9–12 per group) were measured in serum by ELISA. Bars represent mean ± s.d., ^#^p < 0.05 versus Panx1^fl/fl^ mice at the same age, and lines indicate age effect, by 2-way ANOVA.

Aging also led to changes in dynamic and static histomorphometry in the cancellous bone of the lumbar vertebrae (Fig. 5). Specifically, mineral apposition rate and bone formation rate were decreased in 13-month-old mice (Fig. 5A). Further, osteoclast and eroded surface (Fig. 5B) and osteoblast number and surface (Fig. 5C) were decreased in 13-month-old mice compared to 4-month-old mice, independently of Panx1 expression. Consistent with the decrease in osteoblast number and surface, the levels of the circulating markers of bone formation P1NP and alkaline phosphatase were decreased in 13-month-old mice of both genotypes, compared to young mice (Fig. 5D). Conversely, deletion of Panx1 led to a significant increase in bone formation rate in young mice and a decrease in osteoclast surface in aged mice (Fig. 5A,B), without altering the circulating levels of bone formation markers (Fig. 5D). The discrepancies between bone formation measured by dynamic histomorphometry and circulating markers are likely due to the fact that whereas histomorphometry assess the local activity of the osteoblasts, the circulating parameters reflect changes in the whole animal. Indeed, whereas we found increase in MAR and BFR in young pannexin deficient mice in cancellous bone, these parameters were not changed in cortical bone. This lack of higher osteoblast activity in the cortical compartment could have diluted the P1NP and ALP produced by cancellous bone osteoblasts, present in the circulation.

### Reduced expression of osteoclastic genes in bones from aged Panx1^ΔOt^ mice

**Figure 6:**
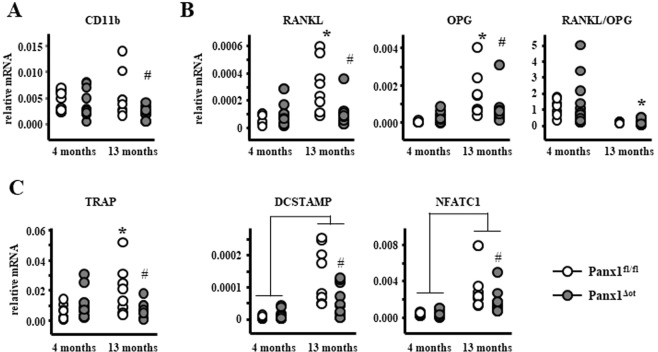
Deletion of Panx1 in osteocytes decreases osteoclastic gene expression in aged 13-month-old female mice. (**A**) CD11b mRNA levels were measured in non-adherent bone marrow cells by qPCR and corrected by GAPDH (n = 9–12 per group). (**B**–**D**) mRNA levels of RANKL and OPG as well as the indicated osteoclast-related genes were measured in bone marrow-flushed long bones by qPCR and corrected by GAPDH (n = 9–12 per group). Bars represent mean ± s.d., ^#^p < 0.05 versus Panx1^fl/fl^ mice at the same age, *p < 0.05 versus 4-month-old mice of the same genotype, and lines indicate age effect, by 2-way ANOVA.

We then examined whether changes in the expression of osteoclast-related genes could explain the histomorphometric changes observed in aged mice. Although there was a genotype effect on the mRNA levels of Itgam (CD11b), an osteoclast precursor marker, post-hoc analysis only showed a significant decrease in CD11b levels in bone marrow cells from aged Panx1^ΔOt^ mice (Fig. 6A), suggesting that deletion of osteocytic Panx1 could lead to a depletion of osteoclast precursors. Further, analysis of cortical bone preparations (without the bone marrow) showed that the levels of *Tnfsf11*, the gene that encodes for the pro-osteoclastogenic cytokine RANKL, and T*nfrsf11b*, the gene that encodes for OPG. a RANKL decoy receptor that inhibits osteoclastogenesis, were increased in cortical bone of aged Panx1^fl/fl^ mice compared to young animals, resulting in no change in the RANKL/OPG ratio (Fig. 6B). Further, RANKL and OPG levels were significantly decreased in aged Panx1^ΔOt^ mice compared to controls at the same age. However, since both RANKL and OPG were decreased, there were no changes in the RANKL/OPG ratio between mice of the two genotypes at 13 months of age, whereas the ratio was lower in the Panx1^ΔOt^ aged compared to young mice. Further, 13-month-aged Panx1^fl/fl^ mice showed higher mRNA levels for tartrate-resistant acid phosphatase (TRAP), an enzyme produced by osteoclasts, and both control and Panx1^ΔOt^ mice showed increased levels of the osteoclastic genes DCSTAMP (dendritic cell-specific transmembrane protein) and NFATC1 (Nuclear factor of activated T-cells, cytoplasmic 1) mRNA compared to young mice. Deletion of Panx1 led to a decrease in these genes in 13-month-old Panx1^ΔOt^ compared to Panx1^fl/fl^ mice (Fig. 6C). This evidence suggests that a potential beneficial effect of Panx1 deletion due to reduced osteoclast number/activity might be obtained in older animals. However, while we could detect an effect at the gene expression level, the number of osteoclasts or the eroded surface were not altered by Panx1 deletion in cortical bone (Fig. 4B).

### Panx1 deletion results in increased lean and muscle mass in female, but not male mice

**Figure 7:**
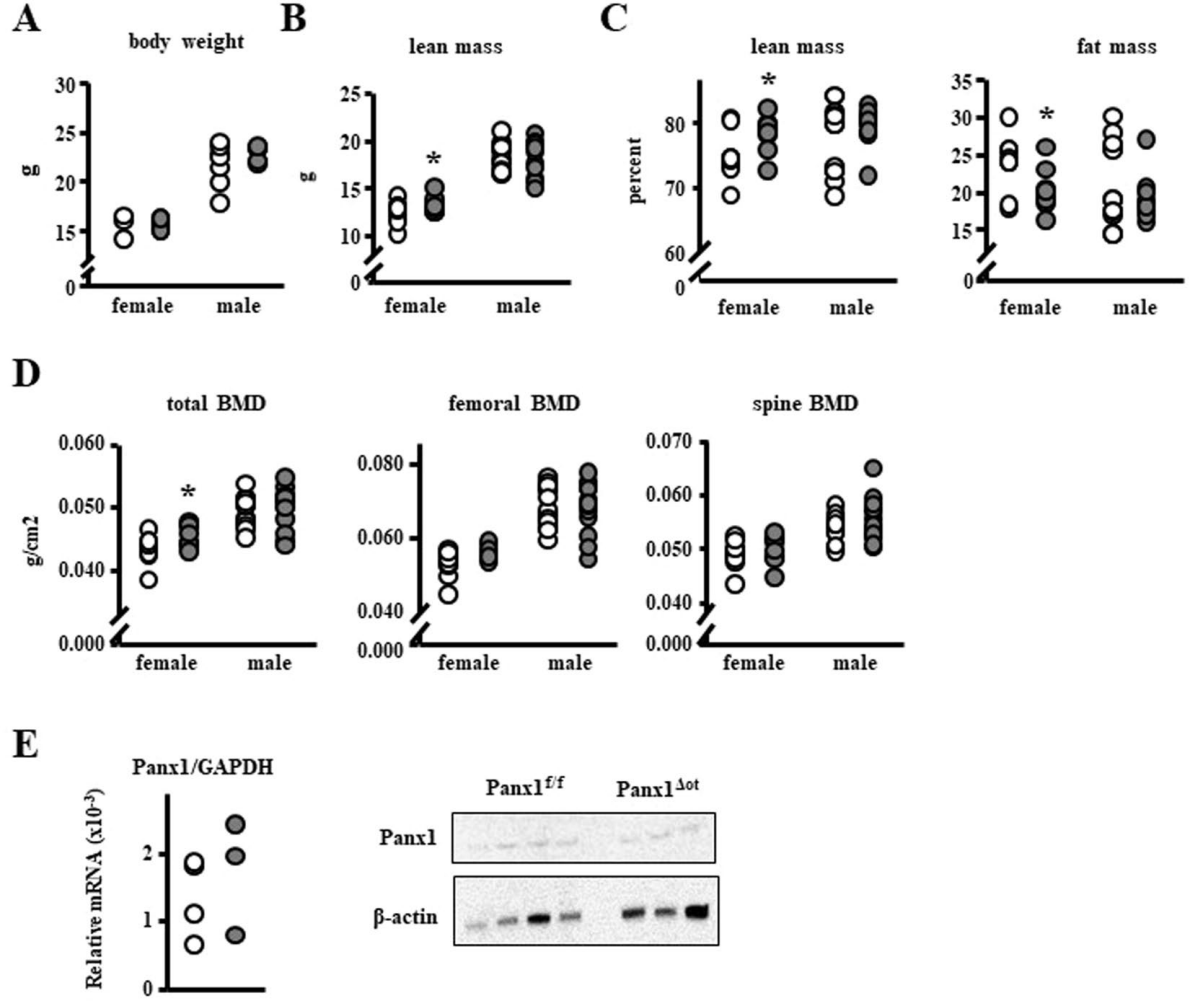
Panx1^Δot^ female mice exhibit increased muscle mass. (**A**–**D**) Body weight **(A)**, total lean body mass (**B**), percent of lean and fat mass (**C**), and BMD (**D**) were measured in 10-week-old female and male mice by DXA (Piximus) (n = 9–13 per group). (**E**) mRNA and protein Panx1 levels were measured in tibialis anterior (TA) muscles by qPCR (corrected by GAPDH, n = 9 and 12 per group), and Western blotting (corrected by β-actin, n = 4 and 3), respectively. Bars represent mean ± s.d., *p < 0.05 versus Panx1^fl/fl^ mice of the same sex, by 2-way ANOVA.

Further analysis of the serial DXA measurement (Fig. 1D) showed a transient increase in total lean body mass of approximately 7% in female Panx1^ΔOt^ compared to Panx1^fl/fl^ mice at 3 and 4 month of age, which was lost by 5 months of age (not shown). We next generated a separate set of mice in order to confirm these changes, and to determine whether male mice also exhibited changes in bone or lean body mass. Mice were analyzed at 10-weeks of age, and the increase in lean body mass was reproduced in female mice, but was not detected in male mice (Fig. 7A). Further, the percent fat mass was only decreased in female mice (Fig. 7B). Similar results were obtained when lean mass was calculated as percentage of total tissue mass (Fig. 7C). We also detected an increase in total BMD in this new cohort of female mice, but not in the males (Fig. 7D). Because muscle is the main component of the lean body mass, and reporter mice revealed that DMP1–8kb-Cre is expressed in skeletal muscle^21^, we then measured Panx1 mRNA and protein levels in tibialis anterior and found no differences between control and Panx1-deficient mice (Fig. 7E).

**Figure 8:**
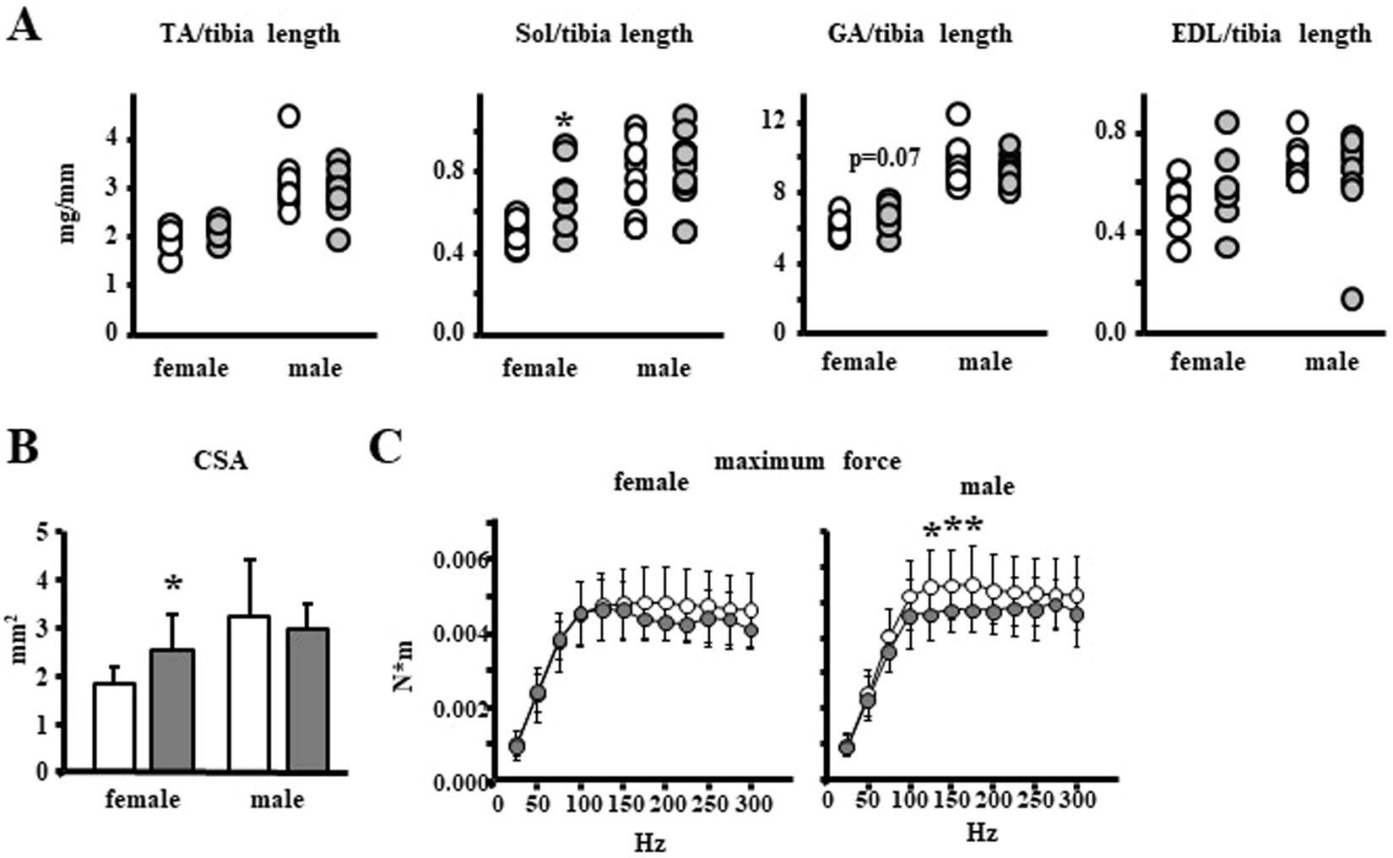
Increased muscle mass in female and decreased muscle strength in male mice lacking Panx1 in osteocytes. (**A**) Muscle weights normalized by tibia length were measured in 10-week old mice (n = 9–12 per group). TA: tibialis anterior, Sol: soleus, GA: gastrocnemius, EDL: extensor digitorum longus muscles. (**B**) Soleus physiological cross-sectional area was calculated [CSA = muscle mass (mg)/[(optimal length) × (fiber to muscle length ratio = 0.72) × (density of muscle = 1.06 mg/mm3)]. (**C**) *In vivo* assessment of maximum force in males and females (n = 9–13 per group). *p < 0.05 versus Panx1^fl/fl^ mice of the same sex, by t-test.

The individual weights of the tibialis anterior, soleus, gastrocnemius and extensor digitorum longus (EDL) muscles were higher in male than in female mice, as expected, but the muscle mass was not altered by osteocytic Panx1 deletion in the males. Further, the weight of the tibialis anterior, gastrocnemius and EDL muscles corrected by tibia length was not different between genotypes for either sex, with only a tendency towards increase in the weight of the gastrocnemius in female mice. However, the soleus muscle weighed 21% more (Fig. 8A) and its cross-sectional area was 38% higher (Fig. 8B) in female Panx1^ΔOt^ compared to control littermates.

We next examined muscle function by conducting an *in vivo* contractility test. To our surprise, we found that whereas deletion of Panx1 in female mice did not alter muscle contractility, there was a significant decrease in this parameter in male mice, consistent with Panx1^ΔOt^ male muscles contracting with lower forces than those of control mice (Fig. 8C). Thus, our data suggests that Panx1 from osteocytes may have a role in skeletal muscle mass and strength, which differs in male versus female mice.

## Discussion

In this study, using a model of conditional Panx1 deletion in osteocytes, we first characterized the contribution of osteocytic Panx1 channels to the adult skeleton. We found that Panx1 deletion resulted in higher bone mass and strength in aged female mice. Removal of Panx1 also reversed the increase in apoptosis-related genes in the 13-month-old mice adding further support to the association between osteocyte apoptosis and reduced bone strength. However, the mechanism that mediates the pro-apoptotic effect of Panx1 channels remains to be determined.

We also found an unexpected effect of Panx1 deletion on lean and muscle mass that was detected only in females, even though Panx1 levels were not changed in skeletal muscle from Panx1^ΔOt^ mice. While there was an overall increase in lean mass in females, only the soleus showed an increase in weight with a corresponding increase in myofiber area. The reason why osteocyte Panx1 function appears to be muscle group specific is presently unknown, but it could involve specific effects on slow contracting muscles like the soleus versus fast contracting muscles such as the EDL, tibialis anterior, and gastrocnemius muscles. This evidence raises the possibility that osteocytes release factors through Panx1 channels that restrain skeletal muscle development in female mice, or that such factors induce subsequent sex-specific events on other pathways or cell types that have the same effect. However, muscle function was not altered by Panx1 deletion in female mice, whereas it was deteriorated in male mice lacking Panx1. This evidence adds to the growing body of research showing that first, osteocytes are able to release molecules that affect skeletal muscle mass and function^22^, and second, that “female” and “male” osteocytes behave differently. Potential factors involved in this bone-muscle interaction are small molecules such as ATP, prostaglandin E2, or nitric oxide, which could be released through Panx1 channels and, therefore, directly impacted by the reduction in Panx1 protein levels; and osteocalcin or TGFβ, bone cell-produced factors known to alter skeletal muscle mass and function and which expression could be indirectly affected by reduction in Panx1 expression or channel function. Further studies are required to identify the osteocyte factors that control skeletal muscle mass and strength, and the mechanisms by which Panx1 channels regulate the expression or release of such factor(s) in a sex- and muscle group-specific fashion. Furthermore, we will investigate whether the sex differences are maintained as the mice age.

The role of pannexin channels in bone is beginning to be revealed^23^. A previous report showed that mice in which Panx1 levels are reduced by 70% in all tissues do not exhibit intra-cortical resorption following fatigue loading, even in the presence of apoptotic osteocytes^6^, suggesting that opened Panx1 channels provide signals that recruit osteoclasts. However, unlike our study in which osteocytic Panx1 deletion led to small, but significant changes in cortical bone structure at 13 months of age, ubiquitous reduction of Panx1 did not result in a phenotype in cortical or cancellous bone in 4–5-month old female mice. Taken together with the current study, these pieces of evidence suggest that the role of Panx1 restraining bone accrual is only apparent as the mice become older or when they are physiologically challenged.

Extracellular ATP induces osteoclast precursor commitment and differentiation into mature cells^24–26^. Thus, reduction of Panx1-mediated osteocyte ATP release could explain the decrease in expression of osteoclastic genes in cortical bone, and the reduced number of osteoclast progenitors in the bone marrow observed in aged Panx1^ΔOt^ mice in our study. This evidence suggests an innate, reduced ability to form osteoclasts in the absence of the channel, as also evidenced by lower fusion of macrophages (which originate from the same precursors as osteoclasts) in cells derived from Panx1 null mice^27^. However, although we found an increase in osteoclast number on the endocortical surface of the femur in the aged Panx1^fl/fl^ mice consistent with the increased expression of the osteoclastic genes in these mice, the reduced expression of these genes in the 13-month-old Panx1^ΔOt^ mice did not translate into changes in osteoclast number on the endocortical surface of the femora (Fig. 4B). This potential discrepancy between gene expression and histomorphometry measurements for osteoclasts in aged mice could be due to the fact that bone preparations used for gene expression might include a combination of cortical and cancellous (in which osteoclast number was decreased, see Fig. 5B) bone. Further, whereas gene expression is corrected by cell number (reflected by the housekeeping gene levels), histomorphometric parameters are corrected by bone surfaces. Another apparent inconsistency is the fact that osteoclast number but not surface, was decreased in cancellous bone. This data suggests that while less osteoclasts are being generated (or more are dying) resulting in lower number of osteoclasts, the ones found on the bone surface are able to spread more, maintaining the percentage of the bone surface covered by osteoclasts similar to that of control littermates. Future studies are needed to determine whether deletion of osteocytic Panx1 increases the ability of osteoclasts to attach to the bone surface. Further, it remains possible that older mice must be used in order to detect a reduction in osteoclast numbers/surface in the absence of Panx1 in osteocytes.

Even though Panx1 deletion has a small effect on femoral cortical bone structure, reversing the decrease in bone area and increasing cortical thickness in the aged mice, a more profound effect of the deletion was found in the mechanical properties of the cortical bone of the femoral mid-diaphysis. Thus, aging induced the expected reduction in both structural and material bone mechanical properties, and deletion of Panx1 was able to reverse the decrease in yield force, displacement to yield, work to yield, yield stress, while increasing strain to yield and resilience. This pattern of differences, most of which are confined to the elastic portion of the mechanical test curve, point to the effects being in the mineral fraction of the tissue (as opposed to the organic/collagen portion)^28^. This evidence, together with the reversal of the increased expression of apoptosis-related genes, adds further support for a role of osteocyte viability in the maintenance of bone mechanical properties. Future studies are needed to further our understanding on the link between osteocyte apoptosis and reduced bone strength.

In summary, our studies suggest previously unrecognized roles of osteocytic Panx1 channels on bone and skeletal muscle. Further, our data suggests that osteocytic Panx1 exerts a role in bone remodeling rather than modeling, affecting bone structure and strength with aging rather than during development. The evidence of the current report raises the possibility of using Panx1 channel inhibitors to ameliorate the deleterious effects of aging in the skeleton.

## Methods

### Mice

Mice with conditional deletion of Panx1 preferentially in osteocytes were generated by breeding Panx1^fl/fl^ mice, provided by Roger J. Thompson (University of Calgary, Calgary, Canada), with mice harboring DMP1-8kb-Cre^18^ in order to obtain homozygous Panx1^fl/fl^; DMP1-8kb-Cre mice (Panx1^Δot^). Mice were fed with regular diet and water *ad libitum*, and maintained on a 12 h light/dark cycle. The animal protocol was approved by the Institutional Animal Care and Use Committee of Indiana University School of Medicine, and animal care was carried out in accordance with institutional guidelines. All studies were conducted in compliance with Public Health Service (PHS) policy, The Animal Welfare Act, “The Guide for the Care and Use of Laboratory Animals”, and other applicable University policies and procedures.

### Whole-mount skeletal staining

Cartilage and mineralized tissue were analyzed in 6-day old newborn mice using alizarin red/alcian blue staining, as previously published^29^.

### Western Blotting analysis

Calvaria protein lysates were prepared as described^30^ with the addition of 1x Halt protease inhibitor cocktail (Thermo Fisher Scientific In., Rockford, IL, USA, cat.#78430) to the lysis buffer. Next, 80 µg of protein were separated on 10% SDS-PAGE gels (GenScript, Piscataway, NJ, USA) and electrotransferred to polyvinylidene difluoride (PVDF) membranes (Bio-Rad Laboratories Inc., Hercules, CA, USA, cat.# 1704157). Membranes were incubated in blocking solution (5% non-fat milk) for 1 hour and probed with primary antibodies in 5% non-fat milk against polyclonal anti-Panx1 (1:100, HPA016930) or anti-β-actin (1:1000, A5316) (Sigma-Aldrich, Saint Louis, MO, USA) overnight at 4 °C. Next day, membranes were incubated with secondary antibodies conjugated with horseradish peroxidase 1:10000 in 5% non-fat milk (Santa Cruz Biotechnology, Santa Cruz, CA, USA) for 1 hour at room temperature. After rinsing with TBS-Tween, the membranes were exposed to an enhanced chemiluminescence Western blotting substrate kit (Pierce Biotechnology Inc., Rockford, IL, USA).

### RNA extraction and real-time PCR (qPCR)

RNA isolation and reverse transcription from marrow-flushed whole bone or bone marrow cells were performed with TRIzol (Invitrogen, Grand Island, NY, USA) and cDNA kit (Applied Biosystems, Foster City, CA, USA), respectively^19^. Gene Expression Assay Mix TaqMan Universal Master Mix and an ABI 7900HT real-time PCR system were used for qPCR. Probes and primers were commercially available (Applied Biosystems, Foster City, CA, USA) or were designed using the Assay Design Center (Roche Applied Science, Indianapolis, IN, USA). Glyceraldehyde 3-phosphate dehydrogenase (GAPDH) was used as the house-keeping gene. Relative expression was determined using the ∆Ct method^29^.

### Bone mineral density (BMD) by dual energy x-ray absorptiometry (DXA)

BMD measurements were performed by DXA using a PIXImus densitometer (G.E. Medical Systems, Lunar Division, Madison, WI, USA)^31^. Measurements included whole body BMD (total BMD, excluding the head and tail), entire femur (femoral BMD) and L1-L6 vertebrae (spinal BMD), fat percentage (total fat body mass (g)/tissue total mass) and lean percentage (total lean body mass (g)/tissue total mass). Calibration was performed before scanning with a standard phantom as recommended by the manufacturer.

### Micro-computed tomography (μCT) analysis

Femora and lumbar vertebrae (L5) were dissected, cleaned of soft tissue, wrapped in PBS-soaked gauze and frozen at −20 °C until imaging^31^. Bones were scanned at 9 μm resolution on a Skyscan 1176 (SkyScan, Kontich, Belgium) following standard procedures, and the recommended nomenclature, symbols, and units^32^.

### Bone histomorphometry

Histomorphometric analysis was performed in femora and lumbar vertebrae (1–3) as previously described^31^. Mice were injected with calcein (30 mg/kg) and Alizarin Red (50 mg/kg) (Sigma Chemical Co, St. Louis, MO, USA), 7 and 2 days before sacrifice, respectively. Dissected tissue was fixed in 10% neutral buffered formalin and processed at the ICMH Histology and Histomorphometry Core. Dynamic histomorphometric analysis was performed on methyl methacrylate embedded unstained femoral mid-diaphysis cross-sections and L1-L3 vertebral longitudinal bone sections using an epifluorescence microscope. Static histomorphometric analysis was performed on TRAPase/Toluidine blue and von Kossa/McNeal stained-sections to visualize osteoclasts and osteoblasts, respectively. Histomorphometric analysis was performed using OsteoMeasure high resolution digital video system (OsteoMetrics Inc., Decatur, GA, USA) using units recommended by the Histomorphometry Nomenclature Committee of the American Society for Bone and Mineral Research (ASBMR)^33^.

### Biomechanical testing

Biomechanical properties were assessed by femoral three-point bending testing following previously published protocols^31^. Briefly, bones were thawed to room temperature and hydrated in 0.9% saline. Femurs were loaded to failure at 2 mm/min with force versus displacement data collected at 10 Hz using a servo-hydraulic test system (TestResources Inc., Shakopee, MN, USA). Material-level properties were determined using μCT anterior–posterior diameter and cross-sectional moment of inertia, as previously described^19^.

### *In vivo* muscle test

*In vivo* muscle strength was measured using the 1305A Whole Mouse/Rat Test System, Aurora Scientific Inc., Aurora, ON, Canada^34^. Data was recorded using the Dynamic Muscle Control/Data Acquisition (DMC) and Dynamic Muscle Control Data Analysis (DMA) programs (Aurora Scientific Inc., Aurora, ON, Canada).

### Statistical analysis

Data were analyzed by using SigmaPlot 13.0 (Systat Software Inc., San Jose, CA, USA). All data were evaluated by t-test or by 2-way ANOVA (Tukey post-hoc test), and values reported as the mean ± standard deviation (s.d.). Table 1 shows the p values for the sex and genotype effects and their interaction. Differences of p ≤ 0.05 were considered significant.

## Data Availability

The datasets generated during and/or analyzed during the current study are available from the corresponding author on reasonable request.
